# Reply to Spiezia et al. Comment on “Gatti et al. Simultaneous Bilateral Reconstruction of Chronic Achilles Tendon Rupture with Flexor Digitorum Longus Transfer and Turndown Flaps: A Case Report and Review of Literature. *J. Clin. Med.* 2026, *15*, 922”

**DOI:** 10.3390/jcm15072667

**Published:** 2026-04-01

**Authors:** Simone Daniel Gatti, Carlo Dante Maria Conti, Agostino Dario Caminita, Judith Waldner, Marco Turati, Giovanni Zatti

**Affiliations:** 1 Orthopedic Department, IRCCS San Gerardo dei Tentori, 20900 Monza, Italy; carloconti60@gmail.com (C.D.M.C.); agostinodario.caminita@irccs-sangerardo.it (A.D.C.); j.waldner@campus.unimib.it (J.W.); marco.turati@unimib.it (M.T.); giovanni.zatti@unimib.it (G.Z.); 2 School of Medicine and Surgery, University of Milano-Bicocca, 20900 Monza, Italy

We would like to thank Dr. Spiezia, Professor Oliva, and Professor Maffulli for their interest in our article and for their thoughtful comments regarding our report on the simultaneous bilateral reconstruction of chronic Achilles tendon rupture [1].

First, we appreciate their clarification regarding the functional consequences of peroneus brevis (PB) tendon transfer. As correctly highlighted by the authors, although measurable reductions in eversion strength have been reported following PB transfer, the clinical relevance of this difference appears limited. Indeed, the available literature has not demonstrated a higher incidence of ankle instability or inversion injuries in patients treated with this technique. PB transfer therefore remains a well-established and reliable option for the reconstruction of chronic Achilles tendon ruptures, with satisfactory long-term outcomes, as reported in their important studies.

In our manuscript, the reference to reduced eversion strength aimed primarily to summarize the biomechanical considerations described in the literature rather than to imply a clinically significant functional deficit. We acknowledge that the clinical impact of this reduction may indeed be minimal in most patients.

Second, we thank the authors for their important clarification regarding the harvesting technique of hamstring autografts. As they correctly emphasize, gracilis and semitendinosus tendons can be harvested with the patient already positioned prone through small incisions at the pes anserinus and more recently through a popliteal approach. This technique effectively avoids the need for intraoperative repositioning [2].

In our institution, however, we are not routinely accustomed to harvesting hamstring tendons with the patient in the prone position. For this reason, in our surgical workflow, the use of hamstring autografts could potentially require additional procedural steps, contributing to increased operative complexity, particularly in the context of a simultaneous bilateral reconstruction. Nevertheless, we fully acknowledge that the prone harvesting technique described by Maffulli and colleagues represents a valuable and elegant solution, and it may certainly be a useful technique to implement in selected cases.

Finally, we agree with the authors regarding the biological rationale for tendon transfers in chronic ruptures, particularly in long-standing injuries where the degeneration of the gastrocnemius–soleus complex may compromise the functional potential of the native musculotendinous unit. In our case, the decision to perform a local tendon transfer rather than using a free graft was guided by this principle, with the aim of introducing an active contractile unit capable of contributing to plantarflexion.

Once again, we thank the authors for their valuable comments and for highlighting important technical considerations that further enrich the discussion on surgical strategies for chronic Achilles tendon rupture.

## References

[1] Spiezia F., Oliva F., Maffulli N. (2026). Comment on Gatti et al. Simultaneous Bilateral Reconstruction of Chronic Achilles Tendon Rupture with Flexor Digitorum Longus Transfer and Turndown Flaps: A Case Report and Review of Literature. *J. Clin. Med.* 2026, *15*, 922. J. Clin. Med..

[2] Rider C.M., Hansen O.B., Drakos M.C. (2021). Hamstring Autograft Applications for Treatment of Achilles Tendon Pathology. Foot Ankle Orthop..

